# Observation of frequency-uncorrelated photon pairs generated by counter-propagating spontaneous parametric down-conversion

**DOI:** 10.1038/s41598-021-92141-y

**Published:** 2021-06-16

**Authors:** Yi-Chen Liu, Dong-Jie Guo, Kun-Qian Ren, Ran Yang, Minghao Shang, Wei Zhou, Xinhui Li, Chang-Wei Sun, Ping Xu, Zhenda Xie, Yan-Xiao Gong, Shi-Ning Zhu

**Affiliations:** 1 National Laboratory of Solid State Microstructures, School of Electronic Science and Engineering, School of Physics, Collaborative Innovation Center of Advanced Microstructures, Nanjing University, Nanjing, 210093 China; 2 Institute for Quantum Information and State Key Laboratory of High Performance Computing, College of Computing, National University of Defense Technology, Changsha, 410073 China

**Keywords:** Quantum optics, Single photons and quantum effects

## Abstract

We report the generation of frequency-uncorrelated photon pairs from counter-propagating spontaneous parametric down-conversion in a periodically-poled KTP waveguide. The joint spectral intensity of photon pairs is characterized by measuring the corresponding stimulated process, namely, the difference frequency generation process. The experimental result shows a clear uncorrelated joint spectrum, where the backward-propagating photon has a narrow bandwidth of 7.46 GHz and the forward-propagating one has a bandwidth of 0.23 THz like the pump light. The heralded single-photon purity estimated through Schmidt decomposition is as high as 0.996, showing a perspective for ultra-purity and narrow-band single-photon generation. Such unique feature results from the backward-wave quasi-phase-matching condition and does not has a strict limitation on the material and working wavelength, thus fascinating its application in photonic quantum technologies.

## Introduction

Spontaneous parametric down-conversion (SPDC) in nonlinear crystals has been a successful technique to generate photon pairs which constitutes a core resource for photonic quantum technologies^1^. However, due to the energy-conserving condition, the photon pairs are usually correlated or entangled in frequency, and consequently, the single-photon state is mixed without frequency information readout or elimination^2^. This feature may bring contamination in many applications involving multiple SPDC sources, or pure single photons^3^.

A straightforward way to eliminate the frequency-correlated information is spectral filtering by a filter with a much narrower bandwidth than the single photons^2^, however, it may reduce the source brightness. One possible solution to this problem is shaping the joint spectrum to produce frequency-uncorrelated photon pairs by adjusting parameters such as crystal length, crystal material and dispersion, phase-matching frequencies, and pump bandwidth^4^. Despite of the fact that several such photon-pair sources have been realized^5–10^, such method relies on modulating the dispersion relationship between the pump and down-conversion photons, i.e., group velocity matching (GVM) condition, and thus has limited choices on the working wavelengths and materials. Another method to produce frequency-uncorrelated photon pairs is to utilize the counter-propagating quasi-phase-matching (QPM) SPDC process^11–14^, where the frequency correlation is eliminated by the narrow-band backward-wave-type phase-matching spectrum function^15–18^, and hence such method can be applied in a large range of nonlinear materials and wavelengths. Due to the counter propagation of the signal and idler photons, an ultra-short poling period in the order of sub-µm is required to satisfy the phase matching condition^19^. Recently, narrow-band^20^ and frequency-uncorrelated^21^ counter-propagating photon pairs generation was demonstrated with the fifth-order QPM. A narrow-band counter-propagating photonic polarization-entanglement source based on the third-order QPM was realized in our lab^22^.

In this paper, we demonstrate an observation of the frequency-uncorrelated photon pairs using the third-order QPM counter-propagating SPDC process in a periodically-poled KTP (PPKTP) waveguide. We measure the joint spectral intensity (JSI) by employing the corresponding stimulated process, namely, the difference frequency generation (DFG) process^23^. This method has been demonstrated to be a rapid and efficient way to characterize the JSI^24–27^. The high precision JSI result exhibits a heralded single-photon purity of 0.996 estimated by Schmidt decomposition. The bandwidth of the backward-propagating photon is as narrow as 7.46 GHz, while the forward-propagating photon has a bandwidth of 0.23 THz similar to the pump light. Such unique feature shows perspective for frequency-multiplexed heralding single-photon generation^28^ as well as other applications in photonic technologies.

## Theory of frequency correlation in counter-propagating SPDC

**Figure 1:**
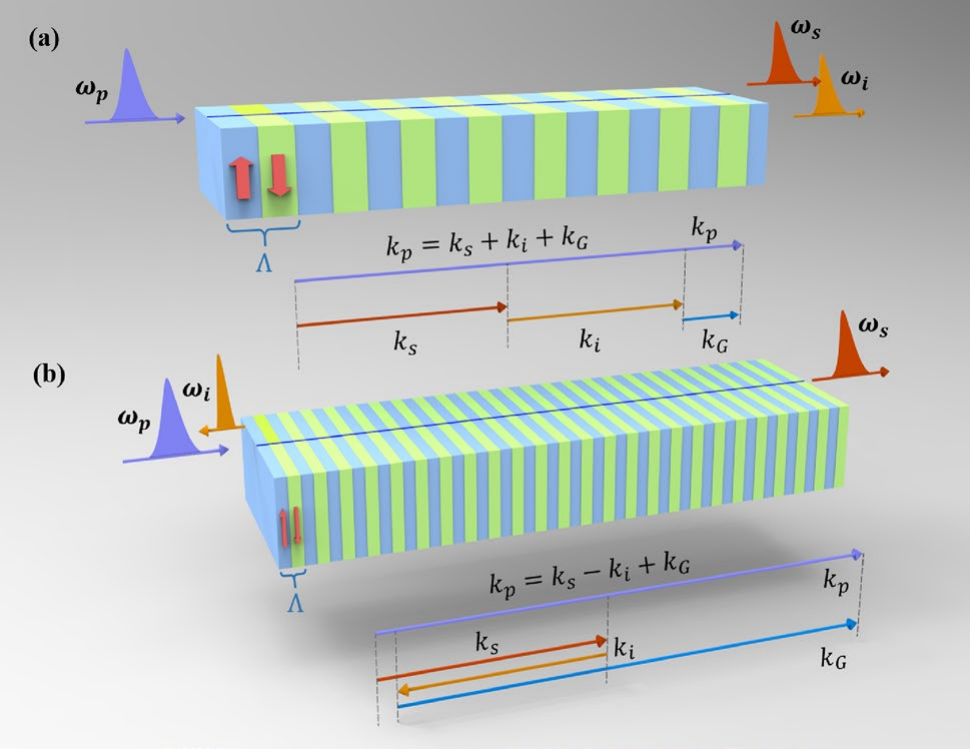
Sketches of photon pair generation and vector diagrams of QPM SPDC processes via conventional co-propagating phase matching (**a**), counter-propagating phase-matching (**b**). Green and blue areas on the crystal are inverted ($-\chi ^{(2)}$) and background positive ($+\chi ^{(2)}$) domains, respectively, with a period of $\lambda$. The purple, red, and oranges arrows represent pump ($k_p$), signal ($k_s$), and idler ($k_i$) wave vectors, respectively, with the reciprocal wave vector $k_G$ denoted by the blue arrow.

The photon-pair state generated from SPDC can be written as^4^

1 $$\begin{aligned} |\psi \rangle =A\int \int d\omega _s d\omega _i f(\omega _s,\omega _i) a^\dag _s(\omega _s)a^\dag _i(\omega _i)|\text {vac}\rangle , \end{aligned}$$

where $|\text {vac}\rangle$ represents the vacuum state, and $a^\dag$ is the creation operator for photons with angular frequency $\omega$, with the subscripts *s* and *i* denoting the signal and idler photons, respectively. The coefficient *A* absorbs all the constants and slowly varying functions of frequency. The spectral property of the photon pairs is determined by the joint spectral amplitude (JSA) given by

2 $$\begin{aligned} f(\omega _s,\omega _i)=\alpha (\omega _s,\omega _i)\phi (\omega _s,\omega _i), \end{aligned}$$

where $\alpha (\omega _s,\omega _i)$ represents the pump spectral function and $\phi (\omega _s,\omega _i)=\text {sinc}(\varDelta k L/2)\text {exp}(-i\varDelta k L/2)$ is the phase-matching function, with *L* denoting the interaction length. In the conventional co-propagating SPDC process, as shown in Fig. 1a, the signal (s) and idler (i) photons propagate in the same direction with the pump (p) photon, where the phase mismatch $\varDelta k$ is written as

3 $$\begin{aligned} \varDelta k=k_p-k_s-k_i-k_G, \end{aligned}$$

where the mth-order reciprocal wave vector $k_G=2\pi m/\Lambda$ with $\Lambda$ denoting the poling period. While in the counter-propagating SPDC process, as shown in Fig. 1b, the signal photons travel in the forward direction along with the pump and the idler photons travel in the opposite direction. Then the phase mismatch is given by

4 $$\begin{aligned} \varDelta k_C=k_p-k_s+k_i-k_{G}. \end{aligned}$$

We define frequency offsets $\delta _j\equiv \Omega _j-\omega _j$, with $j=p,s,i$, where $\Omega _j$ are central frequencies satisfying perfect phase-matching condition $\varDelta k=0$. To analysis the JSA, we expand the phase mismatch to the first order in $\delta _j$, having

5 $$\begin{aligned} \varDelta k&=(k^\prime _p-k^\prime _s)\delta _s+(k^\prime _p-k^\prime _i)\delta _i, \end{aligned}$$

6 $$\begin{aligned} \varDelta k_c&=(k^\prime _p-k^\prime _s)\delta _s+(k^\prime _p+k^\prime _i)\delta _i, \end{aligned}$$

for co-propagating and counter-propagating SPDC processes, respectively. Note that higher-order dispersion can be neglected for the backward-wave-type phase matching^11–18^. Here $k^\prime _j, (j=p,i,s)$ are the inverse of group velocities $u_j$ at central frequencies $\Omega _j$, namely,

7 $$\begin{aligned} k^\prime _j=\left. \frac{\partial k_j}{\partial \omega _j}\right| _{\Omega _j}=\frac{1}{u_j(\Omega _j)}. \end{aligned}$$

In the approximation made in Eqs. (5) and (6), the phase-matching function $\phi (\omega _s,\omega _i)$ is a linear function of $\delta _s$ and $\delta _i$ with a group-velocity angle $\theta$ with respect to the $\omega _i$-axis given by

8 $$\begin{aligned} \theta =&-\arctan \frac{k^\prime _p-k^\prime _s}{k^\prime _p-k^\prime _i}, \end{aligned}$$

9 $$\begin{aligned} \theta _c=&-\arctan \frac{k^\prime _p-k^\prime _s}{k^\prime _p+k^\prime _i}, \end{aligned}$$

for co-propagating and counter-propagating SPDC, respectively. We can see that the angle is related to the group velocities of the pump, signal and idler photons, and thus can be engineered in some specific wavelength ranges^4–10^. However, for the counter-propagating case, $\theta _c$ can keep a small value in a large wavelength range.

The angle $\theta _c$ can be characterized in the temporal domain by introducing the following two characteristic temporal scales^12,13^

10 $$\begin{aligned} \tau _{s}=\frac{L}{2}\left( \frac{1}{u_p}-\frac{1}{u_s}\right) , \end{aligned}$$

11 $$\begin{aligned} \tau _{i}=\frac{L}{2}\left( \frac{1}{u_p}+\frac{1}{u_i}\right) . \end{aligned}$$

The scale $\tau _{s}$ represents the “small” temporal separation between the pump and co-propagating signal waves induced by the group velocity mismatch. The other scale $\tau _{i}$ describes the “large” temporal separation between the pump and counter-propagating idler waves, which is determined by the traveling time through the waveguide of the pulse centers. Therefore, the angle $\theta _c$ given by Eq. (9) can be rewritten as

12 $$\begin{aligned} \theta _c=-\arctan \frac{\tau _{s}}{\tau _{i}}. \end{aligned}$$

In the limit of $\theta _c\rightarrow 0$ the JSA given by Eq. (2) is separable^11–13^, but it is not a sufficient condition due to the role of the pump spectral function. It has been demonstrated^12,13^ that the JSI approaches a factorized form provided that the pump pulse duration $\tau _p$ satisfies the condition of $\tau _{i}\gg \tau _p\gg \tau _{s}$. Note that there is no extra requirement on the specific spectral shape of pump light. Moreover, this condition merely sets a limitation on the temporal scales, without any confinement on the material, dispersion, and working wavelength, provided central frequencies satisfying perfect phase-matching condition of $\varDelta k=0$.

## Experiment and results

In our experiment, we utilize a 10-mm-long PPKTP waveguide with a poling period of $\Lambda =1.3~\upmu$m which can satisfy the third-order QPM condition for type-II counter-propagating SPDC. Fixing the temperature of waveguide at $70^{\circ }$, we expect to obtain the required frequency nondegenerate SPDC, $H_p(784.5\,\text {nm})\rightarrow H_s(1585.5 \,\text {nm})+V_i(1553.08\,\text {nm})$, with H and V denoting the horizontal and vertical polarization, respectively. Based on the temperature-dependent Sellmeier equation^29^, we can obtain the two temporal scales, $\tau _{i}=73\text { ps}$ and $\tau _{s}=0.7\text { ps}$, respectively. Here we set the pump pulse duration $\tau _p=2\text { ps}$ to satisfy the condition of $\tau _{i}\gg \tau _p\gg \tau _{s}$ for frequency-uncorrelated photon pairs generation.

**Figure 2:**
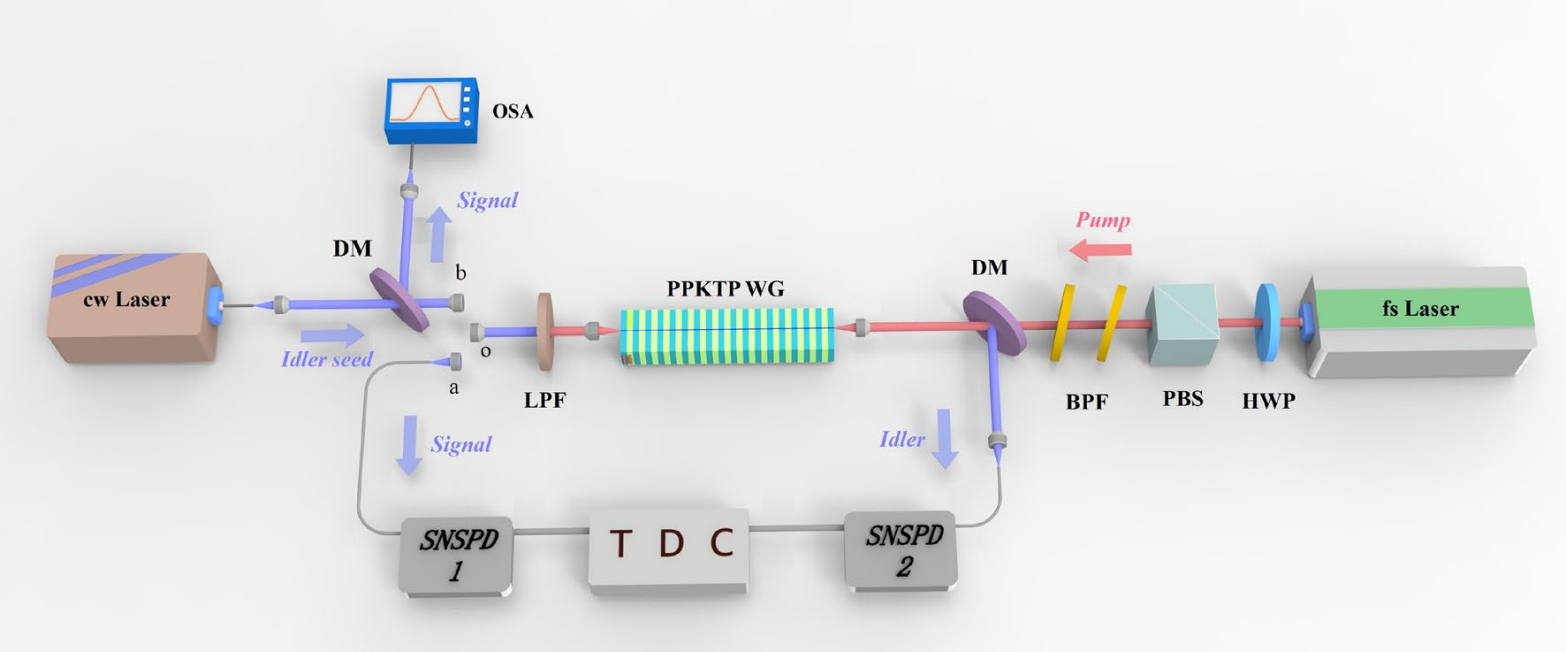
Experimental setup. *HWP* half-wave plate, *PBS* polarizing beam splitter, *DM* dichroic mirror, *BPF* band-pass filter, *LPF* long pass filter, *SNSPD* nanowire superconducting detector, *TDC* time-to-digital converter, *OSA* optical spectrum analyzer, A femtosecond (fs) laser from Ti: Sapphire oscillator serves as the pump light for the SPDC in the PPKTP waveguide (WG). The generated signal photons are coupled into SNSPD 1 by connecting ports a and o, and the idler photons are coupled into SNSPD 2, with the coincidence counts given by TDC. For the DFG process, by connecting ports b and o, a continuous-wave (cw) laser (Santec-550) is used as the idler seed injected together with the fs laser. The signal light is coupled into OSA to measure the spectrum against the wavelength of the cw laser.

**Figure 3:**
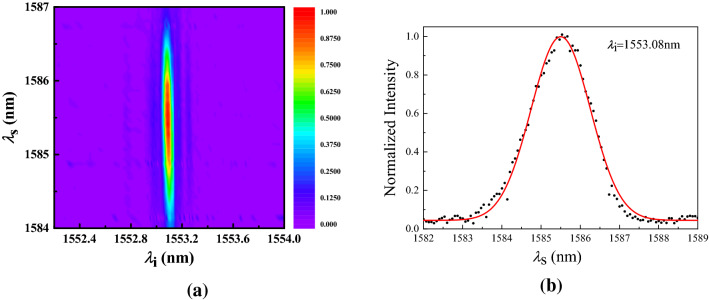
Experimetanl result of the JSI measurement. (**a**) JSI obtained by combining signal spectra measured at 180 idler seed wavelengths. The horizontal resolution depends on the seed linewidth and sweep step, and the step size is set as 0.01 nm. The vertical resolution is 0.065 nm that is determined by the resolution of the optical spectrum analyzer. (**b**) Spectral profile of the signal light measured when the idler seed wavelength is set as 1553.08 nm. The black dots represent the data obtained from the spectrometer and the red solid curve is fitted with a Gaussian function.

The experimental setup is shown in Fig. 2. A femtosecond laser from Ti: Sapphire oscillator with a center wavelength of 784.5 nm first passes a combination of half-wave plate (HWP) and a polarization beam splitter (PBS) to adjust the power, and then is filtered to 2-ps pulses by two band-pass filters. After a dichroic mirror (DM) it is coupled into the waveguide. The forward-propagating signal photon is coupled into a superconducting nanowire single photon detector (SNSPD1) through port a with a long-pass filter (LPF) filtering the pump light. The backward-propagating idler photon is coupled into SNSPD2 after reflected at the DM. The time-to-digital converter (TDC) is used for two-photon coincidence measurement. When the pump power coupled into the waveguide is 11.3 mw, a coincidence counting rate of 870 Hz is measured. Taking into account waveguide-to-fiber coupling efficiency, the transmission loss in the fiber connection from the source to detectors, and the detector efficiency, the total coupling efficiency for signal or idler photon is estimated to be $6\%$, so we can estimate an intrinsic photon pair generation rate to be about $2.1\times 10^4\text { Hz}/\text {mw}$.

To demonstrate the frequency uncorrelation feature of the photon pairs, we measure the JSI^27^, namely, the mode square of the JSA given by Eq. (2). A traditional and direct way to measure the JSI is spectrally resolved single photon coincidence measurements. This method is time consuming and has a low resolution, due to the low generation rate of photon pairs. Here we employ the method of “stimulated emission tomography”^23^ to characterize the JSI, which relies on the relationship between the spontaneous process and its corresponding stimulated process, and is possible with classical detectors, enabling rapid measurement of the JSI and an improved signal-to-noise ratio^24–27^. Here for the SPDC the corresponding process is the DFG process, in which a seed signal or idler pulse is injected together with a pump pulse. As illustrated in Fig. 2, a wavelength-tunable continuous-wave laser (Santec-550) within the idler bandwidth is used as the idler seed to stimulate the emission of signal photons. After a DM it is coupled into the waveguide through port b in the opposite direction of the original pump laser for SPDC. The signal photon is coupled into fiber through port b and then directed into optical spectrum analyzer (OSA) after reflected by the DM. By tuning the idler seed wavelength from 1552.2 to 1554 nm with a step spacing 0.01 nm, we capture the spectrum of signal light by using the OSA with a spectral accuracy of 0.065 nm. The experimental result is shown in Fig. 3a, with a particular example shown in Fig. 3b in the case of the seed wavelength setted as 1553.08 nm.

From the JSI distribution shown in Fig. 3a, we can see that the JSI behaves as an approximate ellipse with its principal axes aligned along $\lambda _s$ and $\lambda _i$. The bandwidth of idler photons is about 0.06 nm, namely, 7.46 GHz, which is consistent with the phase-matching bandwidth. On the other hand, the bandwidth of signal photons is about 2 nm, corresponding to 0.23 THz, which matches well with the pump light bandwidth. The result indicates that the JSI is factorable, with the signal and idler spectrum governed by energy and momentum conservation function, respectively. Our result is in well agreement with the theoretical prediction by Gatti et al.^12,13^. To further evaluate the spectral uncorrelation, we perform the Schmidt decomposition^30^ on the JSI, from which we can estimate the heralded single-photon purity to be 0.996.

## Conclusion

We demonstrate the generation of a frequency-uncorrelated photon pairs using counter-propagating SPDC in a PPKTP waveguide with a poling period on the order of interaction wavelength. By characterizing the corresponding DFG process, we obtain a high-precision JSI image with a heralded single-photon purity of 0.996 estimated by Schmidt decomposition. The underlying physics of our method is the spectral property of the backward-type SPDC phase-matching, and thus this method is not strictly limited by the material, dispersion, and working wavelength. Moreover, here we use the QPM technique to realize the SPDC source, and hence our source is flexible in wavelength choice, provided advanced fabrication techniques^31^. In particular, the backward-propagating idler photon has a narrow bandwidth of 7.46 GHz determined by phase-matching, while the forward-propagating signal photon has a broad bandwidth of 0.23 THz similar to the pump light. The energy-time entanglement between GHz and THz photon pairs may have some unique applications, for instance, the frequency-multiplexed heralding single-photon generation^28^. We hope our approach can stimulate more such investigations.
